# Acid Dentin Lysate Modulates Macrophage Polarization and Osteoclastogenesis In Vitro

**DOI:** 10.3390/ma14226920

**Published:** 2021-11-16

**Authors:** Jila Nasirzade, Zahra Kargarpour, Layla Panahipour, Reinhard Gruber

**Affiliations:** 1 Department of Oral Biology, Dental School, Medical University of Vienna, Sensengasse 2a, 1090 Vienna, Austria; jila.nasirzaderajiri@meduniwien.ac.at (J.N.); zahra.kargarpooresfahani@meduniwien.ac.at (Z.K.); layla.panahipour@meduniwien.ac.at (L.P.); 2 Department of Periodontology, School of Dental Medicine, University of Bern, 3012 Bern, Switzerland; 3 Austrian Cluster for Tissue Regeneration, 1200 Vienna, Austria

**Keywords:** acid dentin lysate, macrophage, osteoclast, inflammation

## Abstract

Dentin prepared from extracted teeth is used as autograft for alveolar bone augmentation. Graft consolidation involves the acid lysis of dentin thereby generating a characteristic paracrine environment. Acid lysate of dentin is mimicking this environment. Acid dentin lysate (ADL) potentially targets hematopoietic cells thereby affecting their differentiation towards macrophages and osteoclasts; however, the question remains if ADL controls macrophage polarization and osteoclastogenesis. Here, we show that ADL reduced lipopolysaccharide (LPS)-induced macrophage polarization of the pro-inflammatory (M1) phenotype, indicated by attenuated Interleukin 1 (IL1), Interleukine 6 (IL6)and cyclooxygenase 2 (COX2) expression. This decrease in M1 macrophages was confirmed by the reduced phosphorylation and nuclear translocation of p65 in the LPS-exposed RAW 264.7 macrophages. Similarly, when RAW 264.7 macrophages were incubated with other agonists of Toll-like receptor (TLR) signaling e.g., FSL1, Polyinosinic-polycytidylic acid High Molecular Weight (Poly (1:C) HMW), Pam3CSK4, and imiquimod, ADL reduced the IL6 expression. We further show herein that ADL decreased osteoclastogenesis indicated by the reduced formation of multinucleated cell expressing cathepsin K and tartrate-resistant acid phosphatase in murine bone marrow cultures. Overall, our results suggest that acid dentin lysate can affect the differentiation of hematopoietic cells to M1 macrophage polarization and a decrease in osteoclastogenesis in bone marrow cultures.

## 1. Introduction

Transplantation of autogenous dentin grafts became a vital strategy in alveolar bone augmentation [1]. First support for this approach came from preclinical studies [2,3,4] and case reports [4] followed by radiological analysis [5,6]. Preclinical research continues to better understand the process of graft consolidation [7,8] and acellular tooth root may even be used as allografts [9]. Clinical studies were performed supporting the use of autogenous tooth roots for augmentation of the alveolar bone [10] and before placing the implant [11]. Autogenous tooth grafting avoided crestal resorption [12] seen in post-extractive implant insertion [13]. Hence, there is increasing evidence for the clinical application of autologous tooth roots for alveolar ridge regeneration. However, the cellular aspects of this clinical approach, in particular the impact of the molecules released during creeping substitution of the dentin graft, is only beginning to be explored. 

One in vitro approach is based on the study of acid dentin lysate (ADL) simulating the acid lysis of the bone-resorbing osteoclasts [14]. In our approach, porcine dentin is grinded and exposed to hydrochloric acid [15,16,17]. The ADL is then processed towards neutralizing the pH and achieving sterility before subjecting it to further exploration. Proteomic analysis of ADL revealed a variety of bioactive molecules including transforming growth factor-β1 (TGF-β1) that maintains its activity after processing [15,16]. RNA sequencing revealed the complex cellular response of mesenchymal cells when exposed to ADL [16,17] and ADL did not impair bone formation in rat calvarial defects [18]. There is, however, a lack of information on how ADL affects the differentiation of hematopoietic cells. 

Hematopoietic cells originating from bone marrow can be differentiated into macrophages using macrophage colony stimulating factor (MCSF) [19]. Once the macrophages are exposed to lipopolysaccharide (LPS) and other toll-like receptor (TLR) agonists they show a massive M1 response indicated by the robust expression of inflammatory clues such as cytokines and chemokine [20]. For the resolution of inflammation, macrophages develop a pro-resolving M2 phenotype that characteristically expresses arginase 1 [21,22] Osteoclastogenesis requires the macrophages responding to nuclear factor kappa-Β ligand (RANKL) to develop the characteristic multinucleated phenotype expressing tartrate-resistant acid phosphatase (TRAP) and cathepsin K [23]. These in vitro models are vital tools to understand the impact of ADL on macrophage polarization and osteoclastogenesis.

Thus, this study intends to determine if ADL influences the polarization of the macrophages and evaluate whether osteoclastogenesis is modulated by ADL.

## 2. Methods

### 2.1. Acid Dentin Lysate (ADL)

Teeth extraction of the whole jaw was performed from pigs following 6 h of scarification (Fleischerei Leopold Hödl, Vienna, Austria). Scarification of the animal was irrespective of the experiment. Periodontal ligaments and soft tissue attachment were removed from the extracted teeth using a surgical blade (Swann-Morton, Sheffield, UK). Teeth were next cleaned using toothbrush and toothpaste. The cleaned teeth underwent a removal of the enamel with a manual grinding and polishing device (Metaserv 2000, Cleveland, OH, USA). Following that, teeth were crushed using a hammer and the remaining pulp tissue was removed by a dental probe (Instrapac, Worksop, UK). Next, the tooth fragments were transferred to HCL 0.1 M and 1.0 M with a ratio of (10% weight/volume), while stirring for overnight at room temperature. Following centrifugation, the supernatant termed as acid dentin lysate (ADL) was neutralized to the physiologic pH. Using filters, ADL was sterilized and after allocating into appropriate tubes, aliquots were stored at −20 °C.

### 2.2. Isolating and Culturing of Murine Bone Marrow-Derived Macrophages and RAW 264.7 Cells

Bagg Albino/c (BALB/c) mice with age of 6- to 8-weeks were bought from Animal Research Laboratories in Himberg, Austria. Bone marrow cells were derived from the femora and tibiae as reported earlier [24]. Concisely, mice were killed, and the femora and tibiae of the mice were detached. Bone marrow cells were cultured at 3.6 × 10^6^ cells/cm^2^ in 24-well plates and left to grow for 5 days in Dulbecco’s Modified Eagle Medium (DMEM, nvitrogen, Grand Island, NY, USA) containing 10% fetal bovine serum, antibiotics and with 20 ng/mL macrophage colony-stimulating factor (M-CSF). RAW 264.7 macrophage-like cells were expanded in growth medium containing Dulbecco’s Modified Eagle Medium (DMEM) having 10% fetal calf serum (FCS) and 1% antibiotic and seeded at 1 × 10^6^ cells/cm^2^ into 24-well plates. Bone marrow macrophages and RAW 264.7 were treated by lipopolysaccharide (LPS) from *Escherichia coli* 0111: B41 (Sigma Aldrich, St. Louis, MO, USA) at 100 ng/mL in the presence and absence of 5% ADL for overnight in a CO_2_ incubator (LabQ, Labconsulting, Vienna, Austria) 37 °C, 5% CO_2_, and 95% humidity. RAW 264.7 cells were stimulated with Pam3CSK4 (Cayman Chemical, Ann Arbor, MI, USA) at 10 μg/mL, poly (1:C) HMW (InvivoGen, Toulouse, France) at 10 μg/mL, FSL-1 (InvivoGen, Toulouse, France) at 20 μg/mL, imiquimod (InvivoGen, Toulouse, France) at 5 μg/mL with and without 5% ADL.

### 2.3. Growing Bone Marrow Hematopoietic-Originated Osteoclasts

Bone marrow cells extracted from femora and tibia of mice were cultured at 3.6 × 10^6^ cells/cm^2^ in 24-well plates and continued to grow for 5 days in Dulbecco’s Modified Eagle Medium (DMEM) having 10% fetal calf serum (FCS) and 1% antibiotic. Receptor activator of nuclear factor kappa B ligand (ProSpec, Ness-Ziona, Israel) at 30 ng/mL, macrophage stimulating colony factor (M-CSF) (ProSpec, Ness-Ziona, Israel) at 20 ng/mL, and TGF-β1 (ProSpec, Ness-Ziona, Israel) at 10 ng/mL were applied to the culture medium to induce osteoclastogenesis. ADL was used at final concentration of 5 percent. Six days later, histochemical staining for tartrate-resistant acid phosphatase (TRAP) was done using the instruction of the manufacturer (Sigma Aldrich, St. Louis, MO, USA). Images were taken by a light microscope with 20X magnification (Oxion fluorescence, Euromex, Arnheim, The Netherlands). 

### 2.4. Live and Dead Staining of RAW 264.7 Cells

RAW 264.7 cells were seeded onto the Millicell^®^ EZ slides (Merck KGaA, Darmstadt, Germany) at 1 × 10^6^ cells/cm^2^. Following incubation of the cells to ADL for overnight prepared in 1.0 M HCl, cells were incubated with Live-DyeTM and Propidium iodide (PI) (Enzo Life Sciences, Lausen, Switzerland) dissolved in a staining buffer for 15 min at 37 °C, 5% CO_2_, and 95% humidity. Immediately after removing the staining solution, photos were taken using Revolve fluorescent microscope with filter blocks for Fluorescein (FITC) and RED (Echo, San Diego, CA, USA).

### 2.5. Reverse Transcription Quantitative Real-Time PCR (RT-qPCR) and Immunoassay

To perform RT-qPCR [25], following overnight incubation total RNA was extracted using the ExtractMe total RNA kit (Blirt S.A., Gdańsk, Poland). Next, complementary DNA (cDND) was synthesized through reverse transcription of the total RNA (LabQ, Labconsulting, Vienna, Austria) and using the cDNA as the template DNA and the appropriate primers, polymerase chain reaction was performed (LabQ, Labconsulting, Vienna, Austria) on a CFX Connect™ Real-Time PCR Detection System (Bio-Rad Laboratories, Hercules, CA, USA). Sequences of the primers were IL1-F: AAGGGTGCTTCCAAACCTTTGAC, IL1-R: ATACTGCCTGCCTGAAGCTCTTGT; IL6-F: GCTACCAAACTGGATATAATCAGGA, IL6-R: CCAGGTAGCTATGGTACTCCAGAA; COX2-F: CAGACAACATAAAACTGCGCCTT, COX2-R: GATACACCTCTCCACCAATGACC; GAPDH-F: AACTTTGGCATTGTGGAAGG, GAPDH-R: GGATGCAGGGATGATGTTCT; ARG1-F: GAATCTGCATGGGCAACC, ARG1-R: GAATCCTGGTACATCTGGGAAC; CCR7-F: AGAGGCTCAAGACCATGACGGA, CCR7-R: TCCAGGACTTGGCTTCGCTGTA; CD163-F: GGCTAGACGAAGTCATCTGCAC, CD163-R: CTTCGTTGGTCAGCCTCAGAGA; CD206-F: GTTCACCTGGAGTGATGGTTCTC, CD206-R: AGGACATGCCAGGGTCACCTTT; cathepsin K-F: TGTATAACGCCACGGCAAA, cathepsin K-R: GGTTCACATTATCACGGTCACA; TRAP-F: AAGCGCAAACGGTAGTAAGG, TRAP-R: CGTCTCTGCACAGATTGCAT. The amount of each specific mRNA was counted through normalizing to the housekeeping gene Glyceraldehyde 3-phosphate dehydrogenase (GAPDH) by the ΔΔCt method. Supernatants of each well representing an independent sample were measured for IL6 secretion by immunoassay based on the manufacturer’s instruction (R&D Systems, Minneapolis, MN, USA). RT-qPCR data are depicted compared to the unstimulated control, which was assumed, as 1.0 in all the analysis. In IL6 enzyme-linked immunosorbent assay (ELISA) the absolute value of secreted protein (ng/mL) from the cells were stated.

### 2.6. Immunofluorescence Analysis

The immunofluorescent examination of intracellular translocation of NF-κB p65 to the nucleus was implemented in RAW 264.7 cells seeded into Millicell^®^ EZ slides (Merck KGaA, Darmstadt, Germany) at 1 × 10^6^ cells/cm^2^. Cells were first serum deprived overnight and next treated with ADL 1.0 M for 30 min. Afterwards. the cells were stimulated by LPS from *Escherichia coli* 0111: B41 (Sigma Aldrich) for another 30 min. The cells were fixed using 4% paraformaldehyde, and blocked with 1% bovine serum albumin (Sigma Aldrich, St. Louis, MO, USA). NF-κB p65 antibody (IgG, 1:800, Cell Signaling Technology, #8242, Cambridge, UK) was added to the cells at 4 °C for overnight. Secondary detection antibody was with the goat anti-rabbit Alexa 488 secondary (1:1000, Cell Signaling Technology, #4412, Cambridge, UK) and Fluoromount-G^TM^, with 4′,6-diamidino-2-phenylindole (DAPI) (Invitrogen, Carlsbad, CA, USA). Images were taken using Revolve fluorescent microscope (Echo) with filter blocks for FITC and DAPI.

### 2.7. Western Blot

RAW 264.7 cells with number of 1 × 10^6^ cells/cm^2^ were seeded into 12-well plates. The next day, cells were treated by ADL for 30 min and then they were exposed to LPS for another 30 min. Lysates of the cells were prepared using SDS buffer containing protease and phosphatase inhibitors (cOmplete ULTRA Tablets and PhosSTOP; Roche, Mannheim, Germany). Followed by that, lysates were divided by SDS-polyacrylamide gel electrophoresis (SDS-PAGE) and transferred onto Polyvinylidene Fluoride (PVDF) membranes (Roche Diagnostics, Mannheim, Germany). Membranes were blocked and the binding of the first antibody NF-κB p65 antibodies (IgG, 1:1000, Cell Signaling Technology, #8242, Cambridge, UK), phospho-p65 antibody (IgG, 1:1000, Cell Signaling Technology, #3031), was identified with the second antibody labelled with horseradish peroxidase anti-rabbit (IgG, 1:10,000, Cell Signaling Technology, #7074). Following incubation by the Clarity Western ECL Substrate (Bio-Rad Laboratories, Inc., Hercules, CA, USA) chemiluminescence signals were envisioned with the ChemiDoc imaging system (Bio-Rad Laboratories, Hercules, CA, USA).

### 2.8. Statistical Analysis

The experiments were repeated three times. Every single data point belongs to an individual experiment, with data points being independently achieved from the teeth of different pigs. Statistical analysis was based on Mann–Whitney U and Kruskal–Wallis tests. Analyses were performed using Prism v8 (GraphPad Software, 2018, La Jolla, CA, USA). Significance was set at *p* < 0.05.

## 3. Results

### 3.1. ADL Reduces LPS-Induced M1 Polarization in Bone Marrow-Derived Macrophages and RAW 264.7 Cells

**Figure 1:**
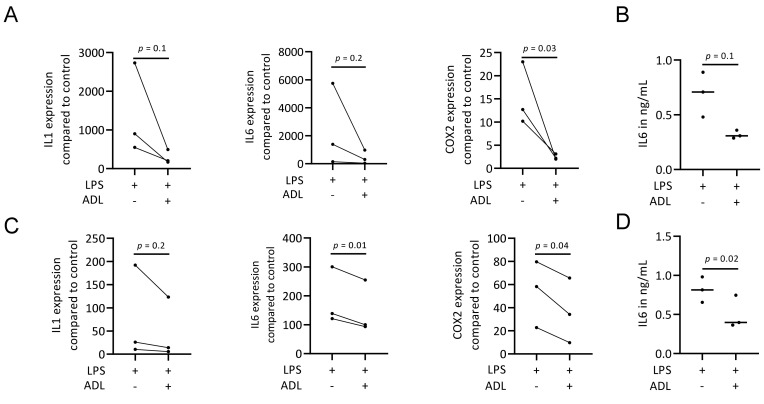
ADL reduces M1 polarization of murine bone marrow-derived macrophages and RAW 264.7 cells. Bone marrow-derived macrophages and RAW 264.7 cells were exposed to LPS with and without ADL 0.1 M for overnight. (**A**–**D**) represent RT-PCR analysis and immunosorbent assay of inflammatory markers in murine bone marrow-derived macrophages and RAW 264.7 cells, respectively. (**C**,**D**) show in RAW 264.7 cells. Statistical analysis was based on a Mann-Whitney U test, and *p* values are indicated.

**Figure 2:**
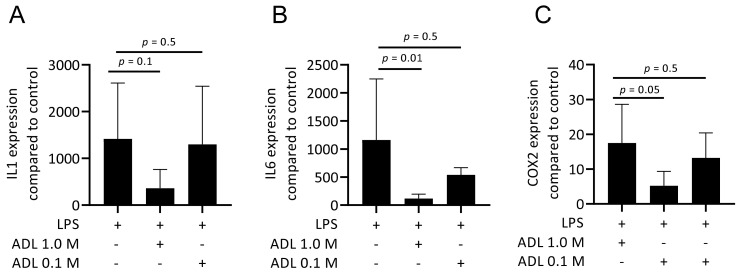
ADL produced from HCl 1.0 M poses a higher anti-inflammatory effect in comparison to ADL prepared from 0.1 M HCl. Data shows RT-PCR analysis of the inflammatory markers (**A**) IL1, (**B**) IL6, and (**C**) COX2. Statistical analysis is based on one-way ANOVA multiple comparison and *p* values show a comparison of each group to LPS.

To evaluate the effect of ADL on polarization of murine bone marrow-derived macrophages and RAW 264.7 cells towards a pro-inflammatory (M1) phenotype, cells were exposed to LPS with and without ADL prepared at 0.1 M HCl. Real-Time Quantitative Polymerase Chain Reaction analysis of IL1, IL6 and COX2, representative of the M1 phenotype and immunosorbent assay of IL6 protein level were conducted. ADL decreased the expression of the pro-inflammatory marker genes and IL6 protein in primary macrophages and (Figure 1A,B). This observation was also observed in RAW 264.7 cells (Figure 1C,D). When comparing ADL prepared with 0.1 and 1.0 M HCl, ADL from 1.0 M was more potent to reduce expression of the inflammatory genes in RAW 264.7 cells (Figure 2) and bone marrow derived stem cells (Supplementary Table S1). Accordingly, no changes were observed in expression of cell surface markers of M1 phenotype CCR7. There was an increase of M2 phenotype markers arginase-1 (Supplementary Figure S2), CD163 and CD206 (Supplementary Figure S2). These results show attenuating effects of ADL on the M1 polarization of macrophages. 

### 3.2. ADL Exposure Maintains Viability of RAW 264.7 Cells

**Figure 3:**
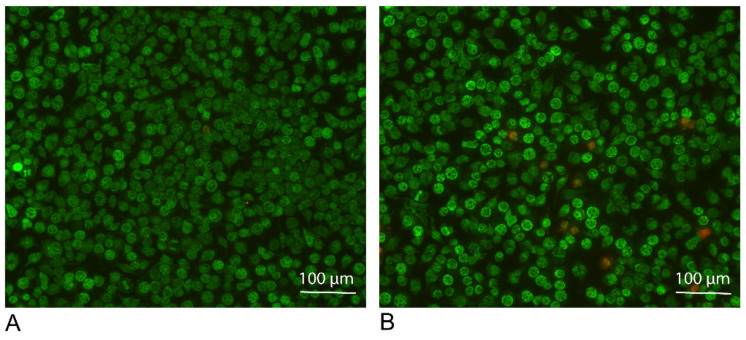
ADL maintains viability of the RAW 264.7 cells. RAW 264.7 Cells were incubated (**A**) without or (**B**) with ADL 1.0 M for overnight. Live and dead staining of the cells were performed using Live-Dye^TM^ and PI for staining of the live (green) and dead (red) cells, respectively.

To make sure that the ADL prepared with 1.0 M HCl is not toxic for the cells, live and dead staining was performed. Results of live and dead staining showed viability maintenance of the cells in the presence of ADL 1.0 M. There is an obvious stronger staining of the cells exposed to ADL. This represents a higher esterase activity in the presence of ADL comparing to the untreated control, resulting in a further rendering of the fluorescent dye, suggesting that cells are viable in the presence of ADL prepared from 1.0 M HCl (Figure 3). Thus, further analysis was made using ADL prepared in 1.0 M HCl.

### 3.3. ADL Inhibits LPS-Induced Phosphorylation of NF-κB p65 in RAW 264.7 Cells

**Figure 4:**
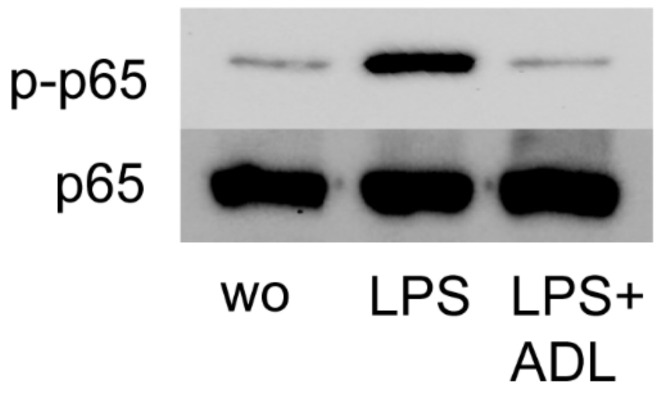
ADL prepared with 1.0 M HCl reduces phosphorylation of NFκB-p65 in RAW 264.7 cells. Western blot analysis revealed the chemiluminescence signals obtained with the phospho-p65 and p65 antibodies.

Activation of NF-κB p65 signaling at the level of phosphorylation is an important indicator of inflammation [26]. To further assess how ADL modulates the inflammatory response, RAW 264.7 cells were exposed to LPS with and without ADL. Western blot analysis revealed that ADL apparently reduces the NF-κB p65 phosphorylation level (Figure 4). Therefore, and consistent with the findings from gene expression analysis, ADL reduces M1 polarization of macrophages at the level of NF-κBp65 phosphorylation.

### 3.4. ADL Decreases LPS-Induced Translocation of NF-κB p65 in RAW 264.7 Cells

**Figure 5:**
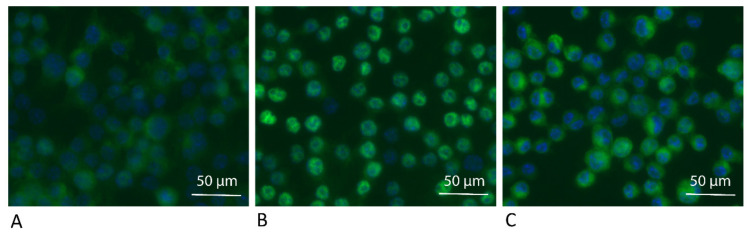
ADL reduces intracellular translocation of NF-κB P65 in RAW 264.7 cells. RAW264.7 cells were treated as (**A**) Unstimulated control, (**B**) LPS, (**C**) LPS + ADL 1.0 M. Immunostaining revealed the green fluorescence signals obtained with the p65 antibody. Nuclear staining with DAPI appears blue.

Translocation of NF-κB p65 from cytoplasm to nucleus is increased through polarization of macrophages towards the pro-inflammatory M1 phenotype [27]. To assess whether this process can be regulated by ADL, immunostaining of the RAW 264.7 cells exposed to LPS alone or in combination with ADL was performed. In line with the results from Western blot analysis, immunostaining revealed an obvious reduction of p65 nuclear translocation in the presence of ADL (Figure 5).

### 3.5. ADL Reduces TLR Agonist-Induced M1 Polarization in RAW 264.7 Cells

**Figure 6:**
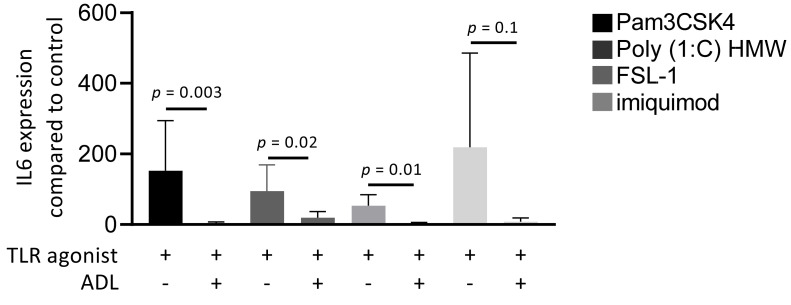
ADL prepared at 1.0 M HCl reduces the inflammatory response of different TLRs. RAW 264.7 cells were exposed to Pam3CSK4, Poly (1:C) HMW, FSL-1, and imiquimod in the presence and absence of ADL, overnight. Data represents RT-PCR analysis of IL6. Statistical analysis was based on a Mann-Whitney U test, and *p* values are indicated.

Toll-like receptors (TLRs) initiate an NF-κB-dependent inflammatory signal when recognizing pathogens [28]. To understand which members of TLR family are regulated by ADL, cells were exposed to Pam3CSK4 (TLR2/TLR1 agonist), Poly (1:C) HMW (TLR3 agonist), FSL-1 (TLR2/TLR6 agonist), and imiquimod (TLR7 agonist), either alone or in combination with ADL. Evaluation of IL6 gene expression revealed that ADL reduces the inflammatory response to all four members of TLR family in RAW 264.7 cells (Figure 6).

### 3.6. ADL Decreases Osteoclastogenesis of Murine Bone Marrow-Derived Macrophages

**Figure 7:**
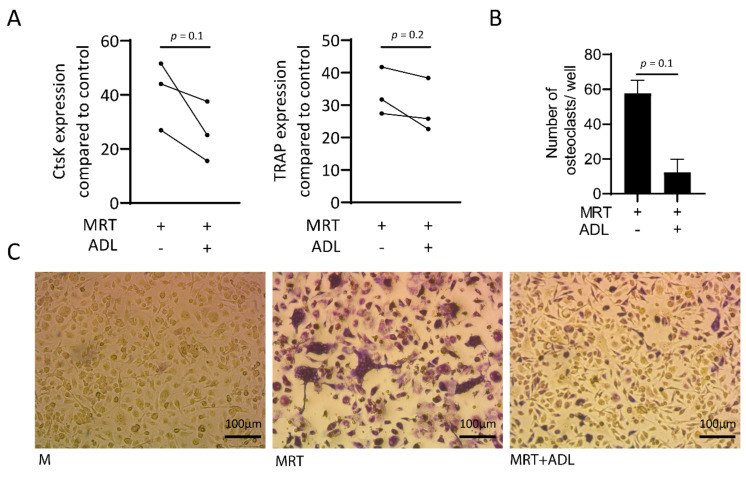
ADL suppresses osteoclastogenesis. Real-Time Quantitative Polymerase Chain Reaction analysis showed reduction in the (**A**) expression of CtsK and tartrate-resistant acid phosphatase (TRAP) and (**B**,**C**) number of multinucleated cells stained positive for TRAP. Statistical analysis was based on a Mann-Whitney U test, and *P* values are indicated. M and MRT stand for macrophage colony stimulating factor (MCSF) and MCSF + receptor activator of nuclear factor kappa beta (RANKL) + transforming growth factor beta (TGF-β), respectively.

Remodeling is one of the critical properties of biomaterials which is executed by osteoclasts [29]. To find out whether ADL effects the process of osteoclastogenesis, Real-Time Quantitative Polymerase Chain Reaction analysis of cathepsin K (CtsK) and tartrate-resistant acid phosphatase (TRAP) together with staining of the multinucleated cells positive for TRAP were conducted. Results showed, although not significant decreased expression of CtsK and TRAP (Figure 7A) and a reduction in number of the cells staining positive for TRAP (Figure 7B) and a higher magnification is provided as Supplementary Figure S1. Taken together, ADL has inhibitory effects on in vitro osteoclastogenesis.

## 4. Discussion

There is a growing evidence in using dentin for extraction socket augmentation and staged implant placement [2,3,4,5,6,7,8,10,11,30]. Graft consolidation is a result of both resorption and formation of the graft [31], thus the release of growth factors from dentin is expected to occur [32]. Odontoclasts, like osteoclasts, provide an acidic environment and the released dentin-derived molecules modify the cells’ microenvironment [33,34,35]. In support of this assumption, we show here that ADL downregulates a TLR-mediated inflammatory response of the bone marrow-derived macrophages and RAW 264.7 cells. On the other hand, ADL increased the expression of arginase-1 in bone marrow-derived macrophages and RAW 264.7 cells as a marker of the pro-resolving (M2) phenotype differentiation [36]. There was also a weak increase of M2 markers CD163 (data not shown) and CD206 (Supplementary Figure S2). Moreover, ADL suppressed osteoclast differentiation of bone marrow-derived hematopoietic cells. Taken together, data from this in vitro pilot study suggest that acid lysate obtained from dentin can attenuate M1 polarization of macrophages and reduce the formation of osteoclast-like cells in vitro. Overall, it seems that dentin holds an intrinsic potential to reduce inflammatory osteolysis and thereby to protect the devital dentin from excessive resorption. 

This study extends previous findings from proteomic analysis and RNA sequencing revealing a potent dentine-derived TGF-β activity that targets the fibroblasts, cells of the mesenchymal lineage [15]. Hematopoietic lineage cells differentiating towards the macrophage and osteoclast lineage are also critically involved in bone regeneration [37,38]. According to our findings that ADL pushed an M1-to-M2 polarization switch and reduced osteoclastogenesis, we can extend our knowledge towards this cell lineage. The decrease in M1 inflammation was rather unexpected because ADL, particular when prepared from uncleaned teeth, has a pro-inflammatory activity in gingival fibroblast [15] and in RAW 264.7 cells [17]. However, in contrast to our previous work we have switched from 0.1 M HCL to 1.0 M HCl to prepare ADL. This new ADL exerts a robust anti-inflammatory and anti-osteoclastogenic activity. At least for in vitro purposes, it is preferred to use 1.0 M HCl to prepare ADL. We assumed that higher molarity of HCl can result in a more substantial release of bioactive molecules from dentin. Obviously, our ADL preparation does not fully reflect the pH 4 that the osteoclasts achieve in the sealed resorption lacunae [39], so our data should be considered a proof-of concept finding. 

Autologous tooth grafts are used for alveolar ridge augmentation. They have shown promising clinical outcomes as comparable to bone grafts with significant increased augmentation area as comparing to control [4]. To investigate the cellular mechanism supporting the regenerative behavior of autogenous tooth grafts we assumed that a part of the regenerative activity is derived by the bioactive molecules embedded in the extracellular matrix of the dentin. Therefore, simulating the acidic environment necessary for osteoclast activity we tried to release the component of the dentin using strong HCl. Even though the clinical relevance remains speculative, the observation that dentin stores an acid-resistant activity that not only lowers the TLR-driven macrophage expression of inflammatory cytokines but also greatly reduces the formation of osteoclasts seems exciting. It can be assumed that this activity is only released when dentin is targeted by osteoclasts, which is usually not the case except at sites of chronic inflammation driving the formation of osteoclasts and in this case odontoclasts. Examples are root resorption during apical periodontitis and orthodontic tooth movement and dental trauma [40,41]. Theoretically, based on our observations, it is the dentin that tries to, if not prevent, at least lowers its own inflammatory osteolysis at sites of chronic inflammation. Thus, dentin can be considered a smart graft that releases an anti-inflammatory/pro-resolving and anti-osteoclastogenic activity on demand. 

Study limitations are manifold. As the first limitation, although not consistent with our prior studies on ADL, we switched from HCl 0.1 M to 1.0 M, which caused a stronger anti-inflammatory activity of ADL. We have normalized the pH, but cannot rule out that small deviations of the pH from neutral, and the increasing concentration of Na^+^ and Cl^−^ ions may exert some M1-to-M2 polarizing activity [42]. Thus, the first question we have not answered is on the characterization of the molecular mechanisms that accounts responsible for the M1-to-M2 polarization switch and the reduced osteoclastogenesis. TGF-β although highly abundant in ADL is not a candidate as recombinant TGF-β has no major impact on M1-to-M2 polarization switch and even increases osteoclastogenesis in vitro [43]. One other candidate molecule could be IL4 known to drive arginase expression and to block osteoclastogenesis, but IL4 was not detected in the proteomic analysis of ADL [15]. Periostin being present in ADL in contrast even pushes macrophage recruitment [15,44]. Another option would be to seek for lipids in ADL that may explain this dual action of ADL on M1-to-M2 polarization switch and the reduced osteoclastogenesis [45], and again, the impact of the high salt concentration of neutralized HCl should not be ruled out [42]. Another limitation is that we have a xenogeneic setting with pig dentin and a murine cell culture system. Thus, future research should determine if human dentin is capable of reducing the LPS-response of CD14-blood derived human macrophages [46] and their differentiation towards human osteoclastogenic cells [47]. Additionally, it would be interesting to investigate the response of induced pluripotent stem cells (IPSCs), which plays a role in regeneration of mineralized tooth component [31] and stem cells derived from oral cavity that are sources of tooth regeneration [32] to ADL. Together, our findings should be considered a primer for future research to better understand how ADL exerts its anti-inflammatory activity—and if this may be of biological or clinical relevance.

Taken together, the present study provides first evidence that acid lysate of porcine dentin can cause a polarization switch of macrophages from the pro-inflammatory M1 to the resolving M2 phenotype, and to reduced osteoclastogenesis in a murine bone marrow system. This research is a pioneer study to better understand the intrinsic activity of mineralized tissues being released by acid lysis—simulating an osteoclast/odontoclast attack at sites of chronic inflammation. The underling molecular mechanisms though remain to be determined.

## Data Availability

Data is contained within the manuscript and Supplementary File.

## Supplementary Materials

The following are available online at https://www.mdpi.com/article/10.3390/ma14226920/s1, Figure S1: ADL suppresses osteoclastogenesis, Table S1: Comparison of anti-inflammatory effect of ADL prepared from HCl 0.1 M and 1.0 M, Figure S2: ADL increases arginase 1 expression in murine bone marrow-derived macrophages and RAW 264.7 cells.
