# Supplementary material for: Acid Dentin Lysate Modulates Macrophage Polarization and Osteoclastogenesis In Vitro
Source: Materials (Basel). 2021 Nov 16;14(22):6920. doi: 10.3390/ma14226920 (PMC8622705; doi:10.3390/ma14226920)
Supplement: Supplementary file 1 [file materials-14-06920-s001.zip › materials-1405569-supplementary.pdf]

materials

Supplementary Materials

Acid Dentin Lysate Modulates Macrophage Polarization and Osteoclastogenesis In Vitro

Jila Nasirzade [1], Zahra Kargarpour [1], Layla Panahipour [1] and Reinhard Gruber [1,2,3,*]

[1] Department of Oral Biology, Dental School, Medical University of Vienna, Sensengasse 2a, 1090 Vienna, Austria; jila.nasirzaderajiri@meduniwien.ac.at (J.N.); zahra.kargarpooresfahani@meduniwien.ac.at (Z.K.); layla.panahipour@meduniwien.ac.at (L.P.)
[2] Department of Periodontology, School of Dental Medicine, University of Bern, 3012 Bern, Switzerland
[3] Austrian Cluster for Tissue Regeneration, 1200 Vienna, Austria
* Correspondence: reinhard.gruber@meduniwien.ac.at; Tel.: +43 (0)699 10718472

Table S1. Comparison of anti-inflammatory effect of ADL prepared from HCl 0.1 M and 1.0 M. ADL prepared from HCl 1.0 M shows a stronger anti-inflammatory effect through suppression of the respective gene expression in bone marrow derived stem cells.

Gene	LPS + ADL 1.0 M/LPS	LPS + ADL 0.1 M/LPS
IL1	0.007	0.201
IL6	0.079	0.415
COX2	0.2	0.3

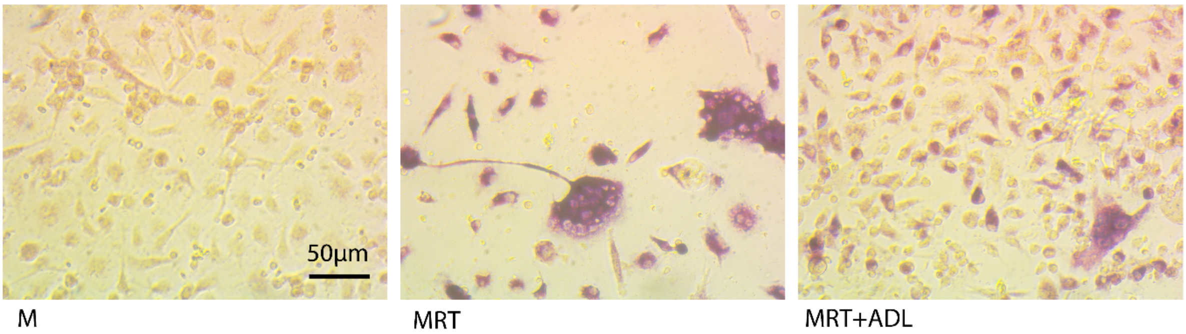

Figure S1. ADL suppresses osteoclastogenesis. Number of multinucleated cells stained positive for TRAP was decreased by ADL. M and MRT stand for MCSF and MCSF + RANKL + TGF-β, respectively.

ADL supports the M2 phenotype of macrophages

The M2 alternative form of the macrophages characteristically express arginase 1 and CD206+ as a cell surface marker. To evaluate whether ADL modulates M2 polarization of the macrophages, expression of CD206+ in murine bone marrow-derived macrophages and arginase-1 in murine bone marrow-derived macrophages and RAW 264.7 cells was determined by RT-PCR analysis. Results revealed an ADL-induced expression of arginase 1 and although not significant results in an increase of CD206+, suggesting that ADL pushes macrophages towards the M2 phenotype (Supplementary Figure S2).

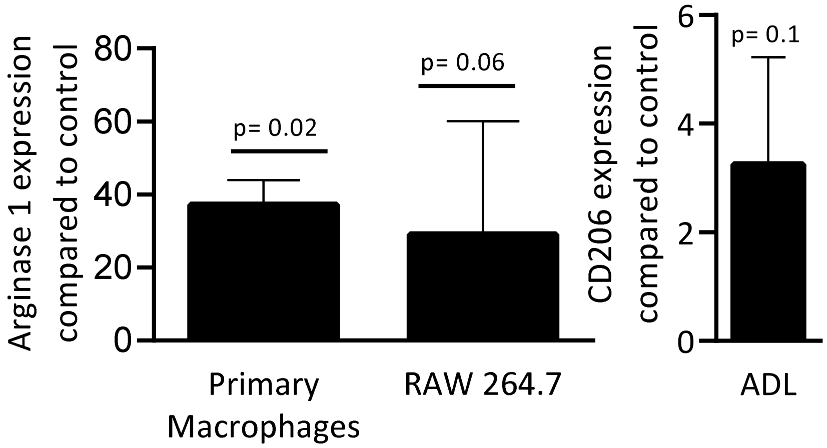

Figure S2. ADL increases arginase 1 expression in murine bone marrow-derived macrophages and RAW 264.7 cells. Bone marrow-derived macrophages and RAW 264.7 cells were exposed to ADL for overnight. Statistical analysis was based on Kruskal-Wallis test, and the P values indicate a comparison of each group to untreated control.
